# Serum biochemical and hematological responses of broiler chickens to herbal-origin anticoccidial supplementation during experimental *Eimeria* infection

**DOI:** 10.3389/fvets.2026.1961791

**Published:** 2026-10-14

**Authors:** Vilma Vilienė, Asta Racevičiūtė-Stupelienė, Daria Murawska, Michal Gesek, Paulius Matusevičius, Zoja Miknienė, Monika Nutautaitė

**Affiliations:** 1 Institute of Animal Rearing Technologies, Veterinary Academy, Lithuanian University of Health Sciences, Kaunas, Lithuania; 2 Department of Animal Welfare and Research, Faculty of Animal Bioengineering, University of Warmia and Mazury, Olsztyn, Poland; 3 Department of Pathological Anatomy, Faculty of Veterinary Medicine, University of Warmia and Mazury, Olsztyn, Poland; 4 Department of Animal Nutrition, Veterinary Academy, Lithuanian University of Health Sciences, Kaunas, Lithuania; 5 Large Animal Clinic, Veterinary Academy, Lithuanian University of Health Sciences, Kaunas, Lithuania

**Keywords:** avian coccidiosis, broiler chickens, *Eimeria* infection, hematology, leukocyte profile, lymphocyte morphology, natural anticoccidials, serum biochemistry

## Abstract

**Introduction:**

Coccidiosis is one of the most economically important diseases in poultry production, prompting increasing interest in natural alternatives to conventional anticoccidial drugs. The aim of this study was to evaluate the biochemical and hematological response of broiler chicken serum to an herbal anticoccidial supplement during experimental *Eimeria* infection.

**Methods:**

A total of 1,200 Ross 308 broilers were assigned to four groups (*n* = 300): non-infected (^−^) and infected (^+^) birds receiving either conventional chemical anticoccidials (UC) or the herbal-origin anticoccidial additive (HA). Blood samples were collected on days 10 and 35 to assess serum biochemical parameters, differential leukocyte profiles and lymphocyte morphology.

**Results:**

Age significantly affected total protein, albumin, AST, GGT, calcium and iron concentrations (*p* < 0.05). A significant group effect was observed for ALT activity (*p* = 0.036) and magnesium concentration (*p* = 0.013), while a significant G x A interaction was found for urea concentration (*p* = 0.040). Significant differences between the groups were also identified band and segmented heterophils, lymphocytes and monocytes, while lymphocyte morphology differed significantly among groups and sampling periods (*p* < 0.05).

**Discussion:**

The study results indicate that the use of a herbal anticoccidial additive was associated with changes in certain serum biochemical and hematological parameters during experimental *Eimeria* infection, without causing adverse changes in the physiological condition of the broilers. These results suggest that herbal anticoccidial supplements may be a promising natural strategy to support the immune response during coccidiosis.

## 1 Introduction

The poultry industry is one of the most significant agricultural subsectors in the world that helps to satisfy the rising demand for meat and eggs (1). However, avian coccidiosis is one of the most significant parasitic diseases in modern poultry farming, causing significant economic losses worldwide (2). This disease is caused by protozoa from the genus *Eimeria*, which damages the intestinal epithelium, disrupts digestive processes and reduces nutrient absorption (3). These violations lead to poor growth and productivity indicators for poultry and increased production costs (2, 4–7). Coccidiosis is estimated to cause significant economic losses to the global poultry industry. In 2016, total financial losses were estimated at around 10.4 billion GBP (2). Other authors have estimated that coccidiosis may cause up to 3 billion USD in economic losses annually, placing a significant financial burden on the poultry industry (8, 9).

Coccidiosis control has traditionally been based on chemical coccidiostats (9–12). However, their long-term use is associated with the development of resistance, the problem of residues in production, and increasing regulatory restrictions (8, 13–19). These challenges encourage the search for alternative, naturally derived solutions. In recent years, increasing attention has been paid to herbal anticoccidial supplements with antimicrobial, antioxidant and immunomodulatory properties (20–26). Although coccidiosis is usually assessed through productivity indicators, *Eimeria* infection causes complex physiological changes in the body. The infection is associated with inflammatory processes, metabolic disorders, oxidative stress and immune system activation (27–29). These processes can be assessed by analyzing blood components, since hematological and immunological parameters sensitively reflect the physiological and immune status of the organism. Previous studies have shown that mixed *Eimeria* infection can cause significant changes in hematological and immunological parameters, reflecting the negative effects of the infection on the host organism. It has also been found that *Eimeria* infection is associated with changes in blood components, which are important in the mechanisms of innate immunity and may be associated with the pathogenic effects of the parasite (30).

Despite the growing interest in herbal anticoccidial additives, most studies have focused mainly on growth, productivity indicators and intestinal health parameters of broilers. Serum biochemical and hematological parameters have also been evaluated in studies of experimental *Eimeria* infection and alternative anticoccidial strategies; however, more detailed data on differential leukocyte responses and, particularly, lymphocyte morphology remain limited (31, 32). Hematological parameters, leukogram, and blood serum biochemical parameters are sensitive indicators of the body's physiological state and the response to infection; therefore, a comprehensive analysis that includes the morphological assessment of lymphocytes can provide additional insight into the biological effects of herbal anticoccidial additives on the organism. In view of this, the aim of this study was to evaluate the biochemical and hematological response of blood serum of broiler chickens to herbal anticoccidial feed additive under conditions of experimental *Eimeria* infection, while also assessing the differential leukocyte count and morphological characteristics of lymphocytes. It was hypothesized that herbal anticoccidials could modulate the physiological response of broilers to experimental *Eimeria* infection, and these changes would be reflected in serum biochemical, hematological, and leukocyte morphological parameters.

## 2 Materials and methods

The study was conducted in accordance with Directive 2010/63/EU of the European Parliament and of the Council on the protection of animals used for scientific purposes and was approved by the Bioethics Committee of the Postgraduate Studies Centre of the Lithuanian University of Health Sciences (permit No. PK042496; 7 November 2022). The study also met the requirements of the State Food and Veterinary Service [No. B6-(1.9)-2625; revised No. B6-(1.9)-852].

### 2.1 Experimental design

A total of 1,200 one-day-old males ROSS 308 broiler chicks were included in the experiment and reared from day 1 to day 35 of age (*n* = 300 birds/group). At the beginning of the study, birds of the same sex and genetic line, obtained from the same hatchery, were randomly allocated to four experimental groups to ensure that the average initial body weight was as similar as possible across the groups. Each group consisted of six replicate pens, with 50 birds in each. All broilers were housed on woodchip litter and managed under standardized husbandry conditions. The broiler chicken stocking density of all groups complied with the requirements of Council Directive 2007/43/EC and did not exceed 33 kg/m^2^. Temperature and lighting regimes were applied according to the recommendations of Aviagen (33).

To prevent cross-contamination between infected and non-infected birds, broilers were housed in two separate poultry houses under identical environmental and management conditions, including temperature, lighting program, ventilation, bedding, and feeding management. Both poultry houses were identical in design, equipment, environmental control systems, management procedures, feeding program, and personnel supervision to minimize potential house-related effects. One poultry house contained the non-infected groups: the chemical anticoccidial control group (UC^−^) receiving conventional coccidiostats (narasin and nicarbazin, 1:1; Elanco US Inc., Greenfield, IN, USA) and the herbal additive group (HA^−^), in which to herbal-origin anticoccidial supplement replaced conventional chemical coccidiostats (500 g/t of feed). The additive consisted of six plant components: *Holarrhena antidysenterica, Ficus religiosa, Berberis aristata, Allium sativum, Embelia ribes*, and *Azadirachta indica*. The second poultry house contained the experimentally infected groups: UC^+^ (infected birds receiving chemical anticoccidials) and HA^+^ [infected birds receiving the herbal-origin supplement (500 g/t of feed)]. Experimental infection with *Eimeria* spp. was administered via drinking water on day 7 of age (Table 1). The control groups were designed to reflect common commercial broiler farming practices, where anticoccidials are routinely used to prevent coccidiosis. Therefore, the study compared a standard chemical anticoccidial program with an herbal alternative, rather than using a completely untreated control group. The experimental dietary treatment and the composition of the feed used were identical to those previously described by Viliene et al. (34) (Section 2.1., Figure 1—Dietary treatments in the feeding trial). This article presents the analysis of serum biochemical and hematological parameters from the same experiment.

**Table 1:** Experimental design and treatment groups.

Group^1^	*Eimeria* infection	Anticoccidial treatment^2^
UC^−^	–	Chemical anticoccidial
HA^−^	–	Herbal-origin additive
UC^+^	+	Chemical anticoccidial
HA^+^	+	Herbal-origin additive

^1^UC, broilers receiving conventional chemical anticoccidials; HA, broilers receiving herbal-origin anticoccidial additives; ^+^, experimentally infected with *Eimeria* spp.; ^−^, non-infected broilers. ^2^The herbal-origin additive consisted of a mixture of dried plant powders (*Holarrhena antidysenterica, Ficus religiosa, Berberis aristata, Allium sativum, Embelia ribes*, and *Azadirachta indica*) supplemented at 500 g/t of feed.

**Figure 1:**
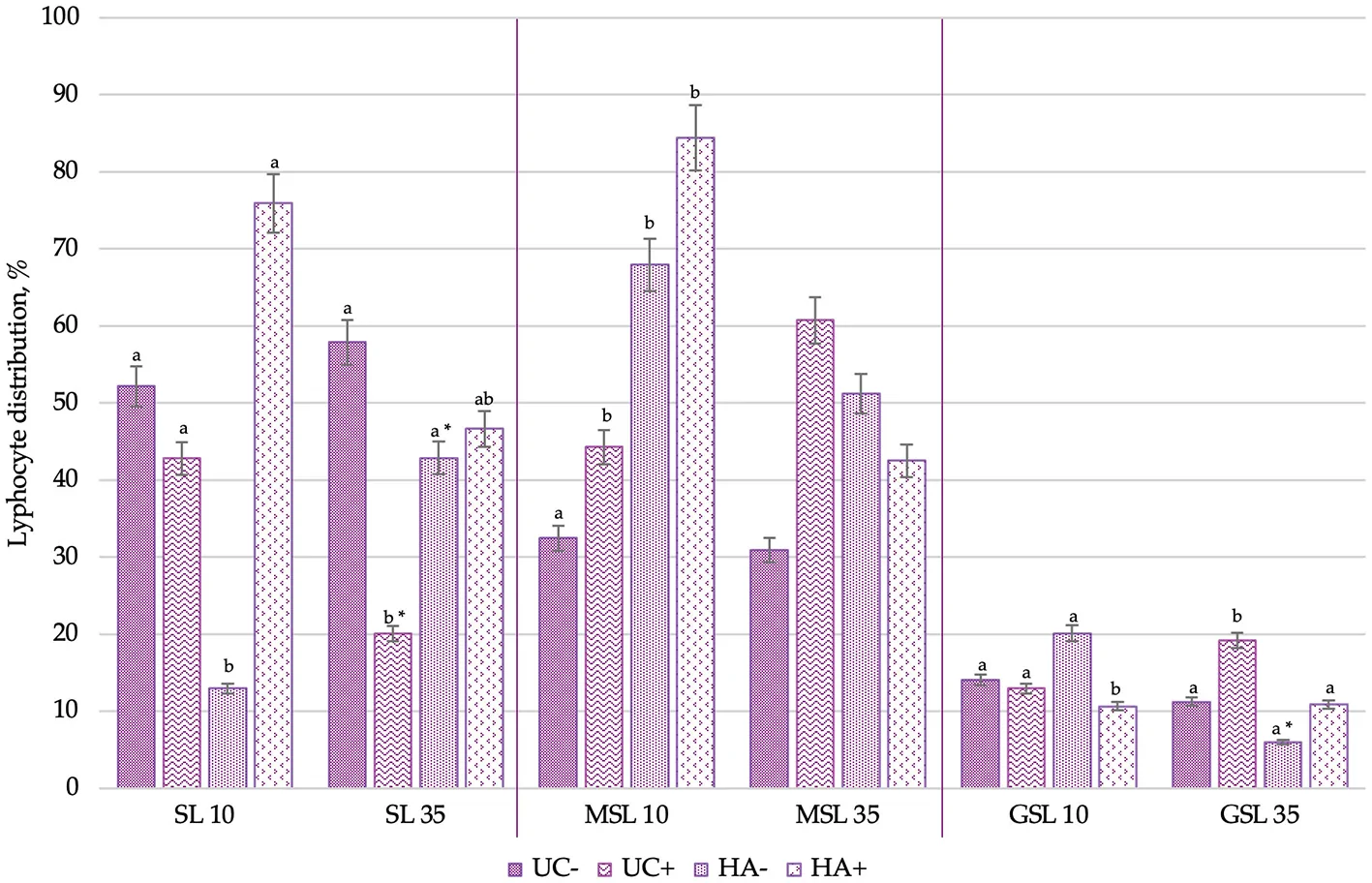
Effects of herbal-origin anticoccidial additives on lymphocyte morphological distribution in broiler chickens at 10 and 35 days of age. SL, small lymphocytes; MSL, medium-sized lymphocytes; GSL, giant lymphocytes. Different letters (a,b) indicate significant differences among experimental groups within the same sampling age (*p* < 0.05). An asterisk (*) indicates a significant difference between sampling ages (10 and 35 days) within the same experimental group (*p* < 0.05).

Experimental infection was established using a standardized preparation of vaccinal attenuated sporulated *Eimeria* oocysts (Paracox-5; Merck Sharp and Dohme Animal Health S.L. MSD Animal Health UK Limited), administered via drinking water. Each bird received one dose (0.004 mL) contained a mixture of sporulated oocysts from five strains: *Eimeria acervulina* [high pathogenicity (HP), 500–650 oocysts], *E. maxima* [current pathogenicity (CP), 200–260 oocysts], *E. maxima* [mild field pathogenicity (MFP), 100–130 oocysts], *E. mitis* (HP, 1,000–1,300 oocysts), and *E. tenella* (HP, 500–650 oocysts).

All groups were provided with balanced compound feed in pellets and supplemented with vitamins and minerals to meet the nutritional needs of growing broilers. The pellet size was adjusted according to the age of the chickens (1–2 mm in the starter period and about 3–3.5 mm in the later phases). Broiler chickens had *ad libitum* access to the feed. The composition and chemical analysis of the feed raw materials used are presented in previously published study by Viliene et al. (34) (Section 2.1., Table 1). The composition and nutrient content of feed ingredients during the pre-starter (1–10 days of age), starter (11–24 days of age), grower (25–30 days of age), and finisher (31–35 days of age) periods. The nutritional value of the feed was calculated according to the recommendations of the Ross 308 Broiler Management Handbook (33).

### 2.2 Blood sample collection and analysis

Blood samples were collected from the broilers on days 10 and 35 of life at 7:00 a.m., before feeding, to assess the early physiological response following *Eimeria* challenge and the final health status of the broilers at the end of the experimental period. At each sampling point, 10 birds per treatment group were randomly selected from different replicate pens to ensure representative sampling. Approximately 1 mL of blood was collected from the brachial vein using a disposable 2 mL syringe fitted with a sterile 23-gauge needle. Individual birds were considered the experimental units for hematological and serum biochemical analyses.

Blood intended for serum biochemical analysis was collected into vacuum tubes without anticoagulant (BD Vacutainer^®^, Becton Dickinson, Crawley, UK). Samples were allowed to clot at room temperature for 30 min and subsequently centrifuged at 3,000 × g for 10 min to obtain serum. The separated serum was transferred into sterile microtubes and stored at −20 °C until biochemical analyses were performed.

Immediately after blood collection, fresh blood smears were prepared from a drop of whole blood, air-dried, and stained using a Diff-Quik staining kit (Everest, Barcelona, Spain). Differential leukocyte evaluation and serum biochemical analyses were performed at the Clinical Laboratory of the Large Animal Clinic, Veterinary Academy, Lithuanian University of Health Sciences.

Differential leukocyte counts were assessed using a light microscope (Nikon Eclipse Ci, Nikon Corporation, Tokyo, Japan) under oil immersion (100 × objective magnification). For each blood smear, 300 leukocytes were counted and classified, and the relative proportions of eosinophils, band heterophils, segmented heterophils, lymphocytes, and monocytes were expressed as percentages. The heterophil-to-lymphocyte (H/L) ratio was calculated as an indicator of physiological stress and immune status. To ensure consistency of microscopic evaluation, all coded blood smears were assessed by the same experienced hematologist, who was unaware of the samples' assignment to experimental groups during the evaluation. In addition, blood smears were examined for lymphocyte morphology (small, medium-sized, and giant lymphocytes), reactive lymphocytes, and cytological inflammatory changes, including vacuolization, toxic granulation, and Döhle-like bodies. The proportions of small, medium-sized, and giant lymphocytes were additionally quantified and expressed as percentages of the total lymphocyte population.

Serum biochemical variables were determined using an automated wet chemistry analyzer (Rx Daytona+, Randox Laboratories Ltd., Crumlin, UK) and commercially available reagents according to the manufacturer's protocols. The analyzed serum parameters included total protein, albumin, alanine aminotransferase (ALT), aspartate aminotransferase (AST), calcium, magnesium, glucose, creatinine, iron, urea, phosphorus, and gamma-glutamyl transferase (GGT).

### 2.3 Statistical analysis

Statistical analyses were performed using STATISTICA software version 13.3 (TIBCO Software Inc., Palo Alto, CA, USA). Data distribution normality was assessed using the Shapiro–Wilk test. For blood analyses, individual birds randomly selected from the experimental pens (*n* = 10 per group at each sampling point) were considered the experimental units.

Blood hematological and biochemical data were analyzed using a two-way analysis of variance (ANOVA) to evaluate the effects of experimental group (UC^−^, UC^+^, HA^−^, HA^+^), age (10 and 35 days), and their interaction (Group × Age). Results are presented as means ± standard error of the mean (SEM). Duncan's multiple range test was used to compare group means, assessing differences between experimental groups at the same sampling time and between sampling periods within each experimental group. Differences were considered statistically significant when *p* < 0.05.

## 3 Results

### 3.1 Serum biochemical parameters

Analysis of serum biochemical parameters showed that age significantly affected most of the evaluated parameters, while the effect of treatment was found only for individual serum biochemical parameters (Table 2).

**Table 2:** Effects of a herbal-origin anticoccidial additive on selected serum biochemical parameters of broiler chickens at 10 and 35 days of age.

Item^1^	Age, days	Group^2, 3, 4^				SEM^5^	*p* value^6^		
		UC^−^	UC^+^	HA^−^	HA^+^		Group	Age	G x A
Total protein, g/L	10	24.27	23.47	23.37	22.53	0.86	0.369	0.000	0.298
	35	^*^29.90	^*^30.27	^*^33.03	^*^30.17				
Albumin, g/L	10	11.18	12.06	11.89	11.55	0.31	0.296	0.000	0.995
	35	^*^13.83	^*^14.63	^*^14.42	^*^13.96				
ALT, U/l	10	5.33^ab^	3.67^bc^	6.33^a^	2.33^c^	0.39	0.036	0.050	0.314
	35	6.33^a^	4.33^b^	6.33^a^	^*^5.67^a^				
AST, U/l	10	125.00	161.67	185.67	169.00	48.85	0.594	0.000	0.425
	35	^*^630.00	^*^624.33	^*^608.00	^*^471.00				
Calcium, mmol/l	10	1.71	2.35	1.83	1.96	0.08	0.104	0.008	0.825
	35	^*^2.23	2.55	^*^2.26	^*^2.47				
Magnesium, mmol/l	10	1.10^b^	1.29^a^	1.00^b^	1.31^a^	0.04	0.013	0.206	0.189
	35	1.13^a^	1.30^a^	0.97^b^	1.01^b^				
Glucose, mmol/l	10	13.44	15.00	13.58	14.45	0.39	0.130	0.343	0.423
	35	13.49	16.90	15.51	13.42				
Creatinine, μmol/l	10	28.53	20.17	26.53	18.25	1.91	0.192	0.609	0.601
	35	22.56	24.17	34.99	19.66				
Iron, μmol/l	10	20.97	24.47	17.40	18.97	1.14	0.960	0.005	0.102
	35	^*^14.20	^*^10.43	15.90	17.17				
Urea, mmol/l	10	0.28^a^	0.42^b^	0.32^ab^	0.25^a^	0.04	0.043	0.214	0.040
	35	0.21^b^	0.12^b^	0.54^a^	0.10^b^				
Phosphorus, mmol/l	10	1.68	2.18	1.59	2.24	0.08	0.052	0.540	0.406
	35	2.05	2.42	1.64	1.94				
GGT, U/l	10	8.37	4.35	8.82	6.28	1.14	0.638	0.000	0.217
	35	^*^14.77	^*^18.35	^*^15.90	^*^13.37				

^1^ALT, alanine aminotransferase; AST, aspartate aminotransferase; GGT, gamma-glutamyl transferase. ^2^UC, broilers receiving conventional chemical anticoccidials; HA, broilers receiving herbal-origin anticoccidial additives; ^+^, experimentally infected with *Eimeria* spp.; ^−^, non-infected broilers. ^3^Different superscript letters (^a−*c*^) within the same row indicate significant differences among experimental groups (*p* < 0.05). ^4^An asterisk (^*^) indicates a significant difference between sampling ages (10 and 35 days) within the same experimental group (*p* < 0.05). ^5^SEM, standard error of the mean.^6^G × A, Group × Age interaction (two-way ANOVA).

Total protein and albumin concentrations increased significantly with age in all experimental groups (*p* < 0.05), but no significant effect of treatment was found on these parameters (*p* > 0.05).

Of the evaluated biochemical parameters related to liver function, treatment significantly affected only ALT activity (*p* = 0.036). On the 10 day of the study, the highest ALT activity was found in the HA^−^ group (6.33 U/L), and the lowest in the HA^+^ group (2.33 U/L; *p* < 0.05). AST and GGT activities were significantly influenced by age (*p* < 0.001), but no effect of treatment was found. On day 35, AST and GGT activity in all groups was significantly higher than on day 10 (*p* < 0.05), but no significant interaction between the treatment and age was observed (*p* > 0.05).

A significant effect of age was found on calcium concentration (*p* = 0.008), whereas no significant effect of the treatment (*p* = 0.104) or interaction between treatment and age (*p* = 0.825) was found.

A significant effect of age on serum iron concentration was observed (*p* = 0.005), however, no significant treatment effect or interaction between treatment and age was found (*p* > 0.05).

Magnesium concentrations were significantly affected by treatment (*p* = 0.013). On the tenth day of the study, the highest magnesium concentrations were found in the UC^+^ and HA^+^ groups (1.29 and 1.31 mmol/L, respectively), and the lowest in the UC^−^ and HA^−^ groups (1.10 and 1.00 mmol/L, respectively; *p* < 0.05). On the 35 day, magnesium concentration was higher in the UC^−^ and UC^+^ groups (1.13 and 1.30 mmol/L, respectively) than in the HA^−^ and HA^+^ groups (0.97 and 1.01 mmol/L, respectively; *p* < 0.05).

A significant group-by-age interaction was found for serum urea concentration (*p* = 0.040). On day 10 of the trial, the highest urea concentration was found in the UC^+^ group (0.42 mmol/L); this was significantly higher than in the UC^−^ (0.28 mmol/L) and HA^+^ (0.25 mmol/L) groups (*p* < 0.05) but did not differ significantly from the HA^−^ group (0.32 mmol/L). On day 35, the highest urea concentration was found in the HA^−^ group (0.54 mmol/L); this was significantly higher than in the UC^−^ (0.21 mmol/L), UC^+^ (0.12 mmol/L), and HA^+^ (0.10 mmol/L) groups (*p* < 0.05), whereas no significant differences were found among the UC^−^, UC^+^, and HA^+^ groups.

### 3.2 Differential leukocyte profile

Differential leukocyte profile analysis showed that dietary treatment significantly affected the percentage of banded heterophils, segmented heterophils, lymphocytes and monocytes, while eosinophils and H/L ratio did not differ significantly (Table 3).

**Table 3:** Effects of the herbal-origin anticoccidial additive on the differential leukocyte profile of broiler chickens at 10 and 35 days of age.

Item, %	Age, days	Group^1, 2, 3^				SEM^4^	*p* value^5^		
		UC^−^	UC^+^	HA^−^	HA^+^		Group	Age	G x A
Eosinophils	10	0.00	0.67	0.33	0.00	0.27	0.711	0.276	0.590
	35	0.33	0.89	0.33	2.00				
Band heterophils	10	3.33^a^	3.67^ab^	4.00^b^	6.67^c^	0.48	0.007	0.834	0.688
	35	3.33^a^	3.67^a^	2.33^b^	7.67^c^				
Segmented heterophils	10	21.33^a^	5.00^b^	25.33^a^	10.67^c^	2.42	0.048	0.025	0.022
	35	36.00^a^	^*^24.00^b^	12.67^c^	24.33^b^				
Lymphocytes	10	75.00^ac^	90.33^b^	69.67^a^	80.33^c^	2.37	0.018	0.016	0.006
	35	59.33^a^	^*^74.67^b^	84.00^c^	64.33^a^				
Monocytes	10	0.33^a^	0.33^a^	0.67^b^	1.78^c^	0.17	0.017	0.852	0.955
	35	0.33^a^	0.67^b^	0.67^b^	1.67^c^				
Heterophil/lymphocyte ratio	10	0.33	0.10	0.42	0.22	0.08	0.452	0.353	0.121
	35	0.66	0.37	0.18	0.50				

^1^UC, broilers receiving conventional chemical anticoccidials; HA, broilers receiving herbal-origin anticoccidial additives; ^+^, experimentally infected with *Eimeria* spp.; ^−^, non-infected broilers. ^2^Different superscript letters (^a−*c*^) within the same row indicate significant differences among experimental groups (*p* < 0.05). ^3^An asterisk (^*^) indicates a significant difference between sampling ages (10 and 35 days) within the same experimental group (*p* < 0.05). ^4^SEM, standard error of the mean.^5^G × A, Group × Age interaction (two-way ANOVA).

The percentage of monocytes differed significantly between experimental groups (*p* = 0.017). The lowest percentage of monocytes was found in the UC^−^ group at both sampling periods, and the highest in the HA^+^ group.

The percentage of banded heterophils was significantly dependent on treatment (*p* = 0.007). The highest percentage was found in the HA^+^ group at both 10 and 35 days of age.

The percentage of segmented heterophils was significantly influenced by group (*p* = 0.048), age (*p* = 0.025) and interaction (*p* = 0.022). In the UC^+^ group, the proportion of segmented heterophils significantly increased from 5.00% to 24.00% (*p* < 0.05).

There was also a significant effect of group (*p* = 0.018), age (*p* = 0.016) and group × age interaction (*p* = 0.006) on the percentage of lymphocytes. In the UC^+^ group, the proportion of lymphocytes significantly decreased from 90.33% to 74.67% (*p* < 0.05).

No significant effect of group, age, or the interaction between group and age was found on the percentage of eosinophils or the heterophil to lymphocyte ratio (*p* > 0.05).

### 3.3 Lymphocyte morphology

The distribution of lymphocyte morphological forms differed significantly between experimental groups and sampling periods (Figure 1).

On the 10 day, the proportion of small lymphocytes was higher in the UC^−^, UC^+^ and HA^+^ groups, and the lowest in the HA^−^ group (*p* < 0.05). On day 35, the proportion of small lymphocytes decreased in the UC^+^ and HA^+^ groups compared to day 10, whereas it increased in the UC^−^ and HA^−^ groups; significant changes were observed in the UC^+^ and HA^−^ groups (*p* < 0.05).

Medium-sized lymphocytes predominated in the HA^+^ group at 10 days of age and in the UC^+^ group at 35 days of age. In all groups, except UC^+^, the proportion of medium-sized lymphocytes decreased from 10 to 35 days.

The proportion of giant lymphocytes at 10 days of age was higher in the UC^−^, UC^+^ and HA^−^ groups than in the HA^+^ group (*p* < 0.05). On the35 day, the highest proportion of giant lymphocytes was found in the UC^+^ group, while it remained significantly lower in the HA^+^ group. Significant age-related changes in lymphocyte morphological forms were found especially in the UC^+^ and HA^−^ groups.

## 4 Discussion

### 4.1 Serum biochemical parameters

In this study, analysis of serum biochemical parameters showed that the largest changes were related to the age of the birds, while the herbal anticoccidial additive significantly affected only individual metabolic parameters. This indicates that most of the assessed biochemical parameters remained stable, and the differences identified were mainly related to physiological growth processes and only partially to the applied feed additive. Similar age-related increases in serum protein concentrations have been reported previously in broiler chickens (8, 35, 36). Although phytogenic compounds have been associated with improved serum protein profiles and enhanced physiological adaptation in poultry (25, 37, 38), no significant treatment effect on total protein or albumin concentrations was observed in the present study.

Serum iron concentrations were significantly higher at 10 days of age than at 35 days in the UC^−^ and UC^+^ groups (*p* < 0.05). This decline may reflect age-related changes in iron metabolism and increased physiological demands associated with rapid growth. Similar alterations have been linked to developmental processes and infectious challenges affecting nutrient utilization and metabolic homeostasis (3, 4, 37, 39).

Biochemical indicators such as AST, ALT, and GGT are commonly used to evaluate metabolic and hepatic status in poultry, although AST activity may also be influenced by skeletal muscle metabolism (25, 40). The lower ALT activity observed in the HA^+^ group may indicate that the use of the herbal origin anticoccidial additives under conditions of experimental *Eimeria* infection was not associated with adverse changes in physiological processes related to liver function. Similar observations have been reported in broilers supplemented with herbal feed additives containing *Allium sativum* and other phytogenic compounds (38, 41–43). Reduced ALT activity has also been described in *Eimeria*-challenged broilers receiving herbal extracts, probiotics, and prebiotics (44), while decreased AST and ALT activities have been observed following supplementation with glycyrrhizin polysaccharides (45). Although a significant group effect regarding liver function-related parameters was observed in this study only for ALT activity, the results do not indicate any adverse effect of the herbal anticoccidial additive on broiler liver function.

Although urea is not the principal end-product of nitrogen metabolism in birds, variations in serum urea concentration may reflect differences in protein turnover, metabolic activity, dietary supplementation, and physiological responses to infectious challenge. Comparable findings have been reported by Mafouo Sonhafouo (46), who observed lower urea concentrations in broiler chickens receiving neem-based supplementation. Similarly, phytogenic additives containing bioactive compounds, including garlic-derived substances, have been associated with modulation of metabolic responses and serum biochemical profiles in poultry exposed to infectious challenges (47, 48). Although the changes in urea concentration detected were small, a significant group x age interaction indicates that the pattern of change in urea concentration among the experimental groups differed depending on the age of the birds.

Magnesium plays an important role in numerous metabolic and enzymatic processes and may be influenced by nutrient absorption and physiological stress (25, 37). Similar alterations in mineral metabolism have been reported in chickens receiving dietary supplements during infectious challenges (3, 8, 13, 49). Our study revealed a significant group effect on magnesium concentration, but no significant interaction between group and age was found. These results indicate differences in magnesium concentration among the experimental groups but do not confirm an age-dependent group response.

In summary, most of the evaluated serum biochemical parameters were influenced more by the birds' age, whereas a significant group effect was observed only for ALT activity and magnesium concentration. A significant interaction between group and age was found only for urea concentration; therefore, changes in these parameters should be interpreted in the context of the overall experimental design and the different anticoccidial strategies.

### 4.2 Differential leukocyte profile

Changes in the differential leukocyte profile reflect the dynamics of the innate and adaptive immune response and are therefore considered sensitive indicators of the immune status of birds during both infection and exposure to various feed additives. In this study, analysis of the leukocyte formula revealed significant differences in the distribution of monocytes, heterophils, and lymphocytes among the experimental groups; this suggests that different anticoccidial strategies under conditions of experimental *Eimeria* infection may have been associated with differences in innate and adaptive immune responses. Monocytes play an important role in the avian innate immune system through phagocytosis, antigen presentation, and participation in inflammatory responses during parasitic infections (30). The higher monocyte proportions observed in birds receiving herbal-origin additives, particularly in the HA^+^ group, may indicate enhanced activation of innate immune mechanisms during *Eimeria* challenge. Similar alterations in immune-related hematological parameters have been reported following supplementation with phytogenic compounds and neem-based products in poultry (46, 50, 51). Nevertheless, leukocyte responses may be influenced by multiple factors, including bird age, infection status, and additive composition. In our study, the highest proportion of monocytes was found in the HA^+^ group, this may indicate differences in the innate immune response between experimental groups under conditions of *Eimeria* infection.

Heterophils are among the primary effector cells of the avian innate immune system and are rapidly recruited during inflammatory and infectious processes, including coccidiosis (25). Increased proportions of band heterophils may indicate activation of early inflammatory responses and enhanced leukocyte mobilization. Similar changes in leukocyte profiles have been described in broiler chickens challenged with *Eimeria* spp. and supplemented with phytogenic feed additives (25, 43). In our study, the higher proportion of band heterophils in the HA^+^ group may indicate a different nature of the early innate immune response under conditions of experimental *Eimeria* infection.

*Eimeria* challenge and dietary treatment influenced heterophil dynamics over time. Because segmented heterophils represent mature inflammatory cells actively involved in host defense, their altered distribution may reflect differences in immune activation and inflammatory status among the experimental groups (8, 25). Similar effects of phytogenic supplementation on immune-related blood parameters have been reported previously in poultry (25, 52). Our results show that changes in heterophile distribution depended not only on the age of the birds but also on the anticoccidial additive applied, which may indicate a different course of the inflammatory response between the experimental groups.

Lymphocytes are central mediators of adaptive immunity and play a key role in protective immune responses against *Eimeria* infection (8, 25). The changes in lymphocyte distribution observed in our study suggest that the use of a herbal origin anticoccidial supplement may have been associated with differences in the regulation of the adaptive immune response under conditions of experimental *Eimeria* infection. The inverse pattern observed between segmented heterophils, and lymphocytes may reflect dynamic changes in immune regulation during growth and infection. Similar alterations in lymphocyte distribution and immune-related hematological indicators have been reported in poultry affected by coccidiosis and in birds receiving phytogenic supplementation (43, 46, 52).

Although the H/L ratio is widely used as an indicator of physiological stress and immune status in poultry, it remained stable in our study; no significant differences in the H/L ratio were found between the experimental groups.

Overall, the observed alterations in monocyte, heterophil, and lymphocyte populations indicate that herbal-origin anticoccidial supplementation may influence leukocyte dynamics during *Eimeria* challenge. The herbal additive used in this study contained plant-derived compounds with reported antioxidant and immunomodulatory properties, including constituents of *Azadirachta indica* and *Allium sativum* (53). Similar immunomodulatory effects have been described for phytogenic additives containing curcumin, thymol, carvacrol, cinnamaldehyde, and other plant-derived bioactive compounds, which have been associated with improved immune responses and enhanced resistance to *Eimeria* infection in broiler chickens (38, 40, 46, 51, 54).

### 4.3 Lymphocyte morphology

The observed changes in the distribution of lymphocyte morphological forms suggest that experimental *Eimeria* infection and the use of the herbal origin anticoccidial additive may have been associated with changes in lymphocyte morphology and immune response dynamics in broiler chickens. Assessment of lymphocyte morphological changes may provide additional information about the activity of immune cells and their response to infection and applied feed additives. Lymphocyte morphology has been proposed as a useful indicator of alterations in immune status and leukocyte activity during infectious and inflammatory conditions. In the present study, the increased proportion of giant lymphocytes observed in infected birds, particularly in the UC^+^ group at 35 days of age, may reflect changes in lymphocyte differentiation and immune cell distribution associated with the host response to *Eimeria* challenge. Similar alterations in leukocyte morphology and hematological profiles have previously been reported in poultry during parasitic infections and following dietary supplementation with phytogenic compounds (50, 55). Our results suggest that an herbal anticoccidial supplement may modulate the distribution of lymphocyte morphological forms during experimental *Eimeria* infection. However, further studies are needed to elucidate the biological significance of these changes and their possible mechanisms of action.

Interestingly, birds receiving the herbal-origin anticoccidial additive under challenge conditions (HA^+^) generally exhibited lower proportions of giant lymphocytes and higher proportions of medium-sized lymphocytes compared with the UC^+^ group. Although the biological significance of these morphological differences remains unclear, the findings suggest that herbal supplementation may influence leukocyte distribution patterns during coccidia infection. Because lymphocyte size alone cannot be considered a direct marker of activation, proliferation, or immune competence, these observations should be interpreted cautiously.

The observed differences may be associated with bioactive compounds present in the herbal-origin additive. Plant-derived substances from *Azadirachta indica, Allium sativum, Holarrhena antidysenterica, Ficus religiosa, Berberis aristata* and *Embelia ribes* contain flavonoids, phenolic compounds, sulfur-containing compounds, and other bioactive molecules that have been associated with antioxidant and immunomodulatory activities (53). Previous studies have shown that phytogenic additives containing curcumin, thymol, carvacrol, and cinnamaldehyde may improve immune responses, intestinal health, and resistance to *Eimeria* infection in broiler chickens (38, 54). Similarly, beneficial effects of neem-based supplementation and herbal extracts on hematological and immune-related parameters have been reported in broilers exposed to coccidia challenge (41, 51, 55).

Overall, the results indicate that *Eimeria* challenge and dietary supplementation influenced the distribution of lymphocyte morphological forms in broiler chickens. While the observed changes suggest altered leukocyte dynamics during infection and growth, further studies are required to clarify the relationship between lymphocyte morphology, immune function, and the biological activity of herbal-origin anticoccidial additives.

### 4.4 Study limitations

Several limitations of the present study should be acknowledged. Infected and non-infected birds were housed in separate poultry houses to prevent cross-contamination; therefore, infection status was partially confounded with the housing environment. Individual birds were used as the experimental units for blood analyses, while the hierarchical structure of birds nested within pens was not incorporated into the statistical model. In addition, the study did not include an infected untreated group receiving no anticoccidial intervention, as the experimental design was intended to compare a conventional chemical anticoccidial strategy with the herbal-origin alternative. Therefore, the observed responses cannot be attributed exclusively to the herbal additive or to *Eimeria* infection itself. Only one herbal blend at a single inclusion rate (500 g/t feed) was evaluated, and quantitative profiling of individual bioactive compounds was not performed. Finally, blood sampling at day 10 represented an early post-challenge response, while the absence of intermediate sampling between days 14 and 21 limited the characterization of the temporal dynamics of hematological and biochemical responses. These limitations should be considered when interpreting the present findings.

## 5 Conclusions

The herbal anticoccidial supplement had limited effect on most blood serum biochemical parameters of broilers chickens; a significant group effect was observed for ALT activity and magnesium concentration, while a significant interaction between group and age was found for urea concentration. In addition, the changes in the proportions of monocytes, heterophils and lymphocytes and the distribution of lymphocyte morphological forms indicated that he use of the supplement may have been associated with differences in the hematological response during experimental *Eimeria* infection. Overall, the results obtained suggest that the evaluated herbal anticoccidial supplement may contribute to the regulation of the immune response, maintaining a stable physiological state of broiler chickens.

## Data Availability

The raw data supporting the conclusions of this article will be made available by the authors, without undue reservation.
